# Clinical risk factors and machine learning-assisted interpretation of ureteral access sheath insertion failure: a multicenter study and meta-analysis

**DOI:** 10.3389/fsurg.2026.1871236

**Published:** 2026-08-26

**Authors:** Yiping Zong, Fan Ouyang, Xiang Gao, Xuzhong Liu, Xinkun Huang, Wei Lu, Peng Han, Wei Zhang, Haibin Hu, Qingyi Zhu, Pei Lu, Zijie Wang, Min Gu, Zhonglei Deng

**Affiliations:** 1 Department of Urology, The Affiliated Yixing Hospital of Jiangsu University, Yixing, China; 2 Department of Urology, The Second Affiliated Hospital of Nanjing Medical University, Nanjing, China; 3 Department of Urology, Huai’an first People’s hospital, Nanjing Medical University, Huai’an, China; 4 Department of Urology, The Affiliated Shuyang Hospital of Xuzhou Medical University, Shuyang, China; 5 Department of Urology, Gaoyou People’s Hospital, Gaoyou, China; 6 Department of Urology, The First Affiliated Hospital of Nanjing Medical University, Nanjing, China

**Keywords:** machine learning, meta-analysis, multi-center, risk factor, ureteral access sheath

## Abstract

**Background:**

In patients undergoing retrograde ureteroscopic lithotripsy, the failure rate of ureteral access sheath insertion is approximately 10%. Temporary double-J stenting with delayed secondary intervention is usually required when insertion fails. However, such cases cannot be reliably identified based on routine preoperative imaging. This study aimed to identify risk factors associated with ureteral access sheath (UAS) insertion failure.

**Methods:**

This retrospective multicenter study included patients who underwent retrograde intrarenal surgery for urolithiasis between January 2025 and December 2025 at six urological centers. Multivariable logistic regression was used as the primary analysis to identify factors associated with UAS insertion failure. An XGBoost model was additionally constructed, and SHapley Additive exPlanations (SHAP) were used to visualize the contribution and direction of included predictors. A systematic review and meta-analysis were further performed to synthesize previous evidence regarding relevant risk factors.

**Results:**

A total of 792 patients were included, of whom 153 patients experienced UAS insertion failure. Multivariable logistic regression identified stone history length, hydronephrosis, age, and stone-size parameters as factors associated with UAS insertion failure. Stone location, particularly proximal ureteral location, showed a risk-increasing pattern in univariable analysis, SHAP interpretation, and meta-analysis. XGBoost and logistic regression showed comparable discrimination in the test cohort, with AUCs of 0.724 and 0.692, respectively. SHAP analysis provided complementary feature-level visualization of predictor contribution and direction. The meta-analysis further supported the association between proximal ureteral location and UAS insertion failure, while the effects of stone history and sex were less consistent.

**Conclusions:**

UAS insertion failure was associated with hydronephrosis, stone-size parameters, and stone history length, with age requiring cautious interpretation. Proximal ureteral stone location may provide additional clinical information when interpreted together with other stone-related factors. SHAP-based visualization and meta-analysis offered complementary evidence to the regression-based findings. These results may support individualized preoperative assessment before attempted placement of a planned 11/13F UAS.

## Introduction

Urolithiasis remains one of the most prevalent urological disorders and a major public health challenge worldwide. Advances in therapeutic technologies have established a systematic treatment framework, including medical expulsive therapy, extracorporeal shock wave lithotripsy (ESWL), ureteroscopic lithotripsy (URS), and percutaneous nephrolithotomy (PCNL). The prevalence remains substantial worldwide: 1%–5% in Asia, 5%–9% in Europe, and 7%–13% in North America (1). Technical innovations such as endoscopic miniaturization, enhanced optical systems and single-use device development have significantly expanded URS applications for both renal and ureteral calculi (2). Among these surgical modalities, retrograde intrarenal surgery (RIRS) has become an important minimally invasive option for renal and proximal ureteral stones due to its minimally invasive nature and higher stone-free rates (3).

The ureteral access sheath (UAS) is an essential tool in modern flexible ureteroscopy, enabling repeated instrument passage through the ureter. Available in various diameters (typically 9.5–14 Fr), the UAS is advanced over a guidewire to the ureter, enhancing irrigation flow, reducing intrarenal pressure, and minimizing friction damage during scope manipulation (4). Current studies comparing UAS-assisted and non-UAS procedures in stone-free rates, operative duration, and complications, demonstrate its safety profile and utility in complex cases (e.g., large/multiple stones or prolonged procedures) (5–7). However, failed UAS placement may require temporary double-J stent placement for 2–4 weeks followed by delayed secondary surgery. Although evidence suggests that preoperative ureteral dilation can improve success rates, the European Association of Urology (EAU) guidelines explicitly discourage routine pre-dilation (2). This clinical dilemma underscores the imperative to identify reliable predictors of UAS insertion failure, which could optimize resource allocation and reduce patient morbidity. Logistic regression remains a standard approach for identifying clinical factors associated with binary surgical outcomes because it provides interpretable odds ratios and confidence intervals (8, 9). Machine-learning approaches have increasingly been applied to clinical prediction modeling and may provide complementary information regarding feature contribution and potential nonlinear relationships (10, 11). Although multiple studies have investigated the risk factors for ureteral access sheath placement failure, the reported findings are inconsistent. The identified risk factors primarily involve patient characteristics, stone-related factors, and imaging findings. Given the substantial heterogeneity and inconsistency of reported findings across these studies, synthesizing the available evidence is necessary to generate more robust and clinically relevant conclusions (12, 13).

This study aimed to identify clinical factors associated with UAS insertion failure in a multicenter cohort of patients undergoing retrograde ureteroscopic lithotripsy. Multivariable logistic regression was used as the primary method for risk-factor analysis, while XGBoost and SHAP analyses were applied as complementary exploratory tools to assess model discrimination and feature contribution. In addition, we conducted a systematic review and meta-analysis to synthesize previous evidence on risk factors for UAS insertion failure.

## Patients and methods

### Study design and statistical analysis

Consecutive patients who underwent retrograde intrarenal surgery with attempted ureteral access sheath placement were retrospectively identified from six participating urological centers according to predefined inclusion and exclusion criteria. The study period spanned from January 2025 to December 2025 across six centers. Case distribution and UAS insertion failure rates were summarized for each participating center to describe center-level representation (Supplementary Table 1).

Univariable and multivariable logistic regression analyses were performed in the full cohort to identify clinical factors associated with UAS insertion failure, with adjusted odds ratios (ORs) and 95% confidence intervals (CIs) reported. A systematic review and meta-analysis were conducted to synthesize previously published evidence and to complement the cohort-based risk-factor analysis. As an exploratory supplementary analysis, an XGBoost model and a logistic regression comparator model were developed using the same candidate predictors (variables with *P* < 0.05 in univariable logistic regression) and the same randomly split training and test cohorts at a 3:1 ratio. XGBoost hyperparameters were tuned in the training cohort using five-fold cross-validation, with AUC as the optimization metric. Model discrimination, calibration, and clinical net benefit were assessed using the area under the receiver operating characteristic curve (AUC), calibration curves, and decision curve analysis (DCA), respectively. The AUCs of the XGBoost and logistic regression comparator models were compared using the DeLong test. SHAP analysis was used to explore feature contribution in the XGBoost model.

### Inclusion and exclusion criteria

UAS insertion failure was defined as failure to advance the planned 11/13F UAS to the target ureteral segment over the guidewire during the initial procedure, resulting in abandonment of sheath placement and delayed secondary surgery after temporary double-J stent placement. This definition focused on failure of planned UAS placement; cases in which initial ureteroscopic access was unsuccessful and therefore precluded UAS placement were also classified as UAS insertion failure.

Patients were eligible for inclusion if they met the following criteria: (1) age 18–80 years; (2) upper urinary tract stones confirmed by CT; and (3) attempted placement of an 11/13F UAS during the initial RIRS procedure.

Exclusions comprised cases aborted due to: (1) active infection (e.g., purulent urine, fever >38.5 °C, or positive urine culture); (2) congenital urological anomalies (horseshoe kidney, duplex ureter system); (3) prior ureteral dilation within 2 months; (4) concomitant additional surgical procedures; (5) missing data.

Collected data included patient demographics and baseline clinical characteristics: height, BMI, gender, smoking history, alcohol use, and histories of hypertension, diabetes, cardiovascular disease and chronic kidney disease. Imaging data from the indexed procedure were also recorded, including side, location, number of stones, major and minor stone diameters, stone density (CT value), and presence of hydronephrosis. Stone-related medical history was also reviewed: previous urolithiasis and its duration, history of urological procedures, and previous UAS placement failure history.

### Search strategy and study selection

Two researchers (Fan Ouyang and Yiping Zong) independently conducted literature searches in PubMed, Web of Science, Embase, Scopus, and the Cochrane Library from database inception to October 31, 2025. The same core search strategy was applied across all databases and adapted only when required by database-specific syntax: (risk factor OR predict* OR inser* OR failure) AND (ureteral sheath OR difficult ureter* OR ureteral access sheath). The search was restricted to English-language publications. The initial search yielded 3,285 records, including 592 from PubMed, 760 from Web of Science, 1,562 from Embase, 175 from Scopus, and 196 from the Cochrane Library.

All retrieved records were imported into EndNote X9 for management and duplicate removal, resulting in 2,062 unique articles. Two investigators independently screened these articles by reviewing titles and abstracts. Discrepancies in inclusion decisions were resolved through discussion with a third reviewer. After this initial screening, 32 articles were retained for full-text assessment. Subsequent full-text review excluded 13 articles due to inappropriate study types (e.g., reviews, meeting abstracts, and editorial materials). Three additional articles were excluded because of an insufficient number of patients in sheath placement failure group, and one article was removed due to non-representative study population. Furthermore, four studies investigating pharmacological interventions (e.g., calcium channel blockers, *α*1-blockers) on sheath placement success rates were excluded. One article was additionally excluded due to unavailability of the full text.

The final selection included 10 articles for qualitative synthesis (14–23). The methodological quality of included studies was assessed using the Newcastle–Ottawa Scale (NOS) by two independent reviewers, with discrepancies resolved through consensus or consultation with a third reviewer. Leave-one-out sensitivity analyses were performed for the main pooled risk factors when sufficient studies were available. Publication bias was not formally assessed because fewer than 10 studies were included in each pooled analysis. The systematic review and meta-analysis component of this study was reported in accordance with the PRISMA statement for the meta-analysis.

### Surgical procedures

All procedures were performed under general anesthesia by multiple surgeons. Under endoscopic guidance, the ureteroscope was advanced into the bladder to identify the target ureteral orifice. A guidewire was inserted into the ureter, and the ureteroscope was navigated retrograde along the wire to assess ureteral patency and stone characteristics. For successful cases, a 11/13F flexible ureteral access sheath (UAS) was placed over the guidewire, followed by holmium:YAG laser lithotripsy (365 µm fiber) to fragment stones to <2 mm; fragments were then extracted using a basket device. Finally, a 6F COOK double-J stent was subsequently deployed on the operative side. In failure cases, the planned 11/13F UAS could not be advanced to the target ureteral segment, and the procedure was terminated after placement of a 5F COOK double-J stent.

## Results

### Basic characteristics of patients

Baseline characteristics of the cohort are summarized in Table 1. A total of 792 patients were included, of whom 153 (19.32%) were classified into the UAS insertion failure group and 639 (80.68%) into the success group.

**Table 1:** Characteristics of included patients.

Variables	Total (*n* = 792)	Success (*n* = 639)	Failure (*n* = 153)	Statistic	*P*
Height, M (Q_1_, Q_3_)	169.00 (162.00, 172.25)	169.00 (162.00, 173.00)	168.00 (162.00, 172.00)	*Z* = −0.58	0.563
BMI, M (Q_1_, Q_3_)	25.10 (22.89, 27.45)	25.10 (23.00, 27.58)	24.97 (22.77, 27.04)	*Z* = −1.13	0.258
Age, M (Q_1_, Q_3_)	53.00 (42.00, 60.25)	52.00 (40.00, 60.00)	54.00 (46.00, 61.00)	*Z* = −2.06	**0**.**039**
Major axis of stones, M (Q_1_, Q_3_)	11.12 (8.25, 14.76)	11.55 (8.79, 15.01)	8.84 (7.04, 12.45)	*Z* = −5.42	**<**.**001**
Minor axis of calculus, M (Q_1_, Q_3_)	7.72 (5.71, 9.78)	8.12 (6.28, 10.18)	6.00 (4.13, 7.83)	*Z* = −8.02	**<**.**001**
CT value, M (Q_1_, Q_3_)	909.00 (657.20, 1,216.00)	914.00 (657.20, 1,216.00)	884.75 (624.00, 1,204.00)	*Z* = −0.45	0.654
Sex, *n* (%)				*χ*^2^ = 0.44	0.507
Female	203 (25.63)	167 (26.13)	36 (23.53)		
Male	589 (74.37)	472 (73.87)	117 (76.47)		
Smoking history, *n* (%)				*χ*^2^ = 3.22	0.073
No	641 (80.93)	525 (82.16)	116 (75.82)		
Yes	151 (19.07)	114 (17.84)	37 (24.18)		
Drinking history, *n* (%)				*χ*^2^ = 0.40	0.526
No	681 (85.98)	547 (85.60)	134 (87.58)		
Yes	111 (14.02)	92 (14.40)	19 (12.42)		
Hypertension, *n* (%)				*χ*^2^ = 3.25	0.071
No	545 (68.81)	449 (70.27)	96 (62.75)		
Yes	247 (31.19)	190 (29.73)	57 (37.25)		
Diabetes, *n* (%)				*χ*^2^ = 1.41	0.235
No	700 (88.38)	569 (89.05)	131 (85.62)		
Yes	92 (11.62)	70 (10.95)	22 (14.38)		
Coronary heart disease, *n* (%)				*χ*^2^ = 0.24	0.626
No	773 (97.60)	625 (97.81)	148 (96.73)		
Yes	19 (2.40)	14 (2.19)	5 (3.27)		
Chronic kidney disease, *n* (%)				*χ*^2^ = 0.21	0.647
No	782 (98.74)	632 (98.90)	150 (98.04)		
Yes	10 (1.26)	7 (1.10)	3 (1.96)		
Stone side, *n* (%)				*χ*^2^ = 4.73	0.094
Left	393 (49.62)	315 (49.30)	78 (50.98)		
Right	336 (42.42)	279 (43.66)	57 (37.25)		
Both	63 (7.95)	45 (7.04)	18 (11.76)		
Stone location, *n* (%)				*χ*^2^ = 6.92	0.074
Renal area	302 (38.13)	257 (40.22)	45 (29.41)		
Proximal ureter	333 (42.05)	256 (40.06)	77 (50.33)		
Mid ureter	89 (11.24)	71 (11.11)	18 (11.76)		
Distal ureter	68 (8.59)	55 (8.61)	13 (8.50)		
Number of stones, *n* (%)				*χ*^2^ = 1.65	0.199
Single	342 (43.18)	283 (44.29)	59 (38.56)		
Multiple	450 (56.82)	356 (55.71)	94 (61.44)		
UAS insertion failure history, *n* (%)				*χ*^2^ = 3.78	0.052
No	752 (94.95)	602 (94.21)	150 (98.04)		
Yes	40 (5.05)	37 (5.79)	3 (1.96)		
Stone history length, *n* (%)				*χ*^2^ = 6.81	**0**.**009**
≤31 days	213 (26.89)	159 (24.88)	54 (35.29)		
>31 days	579 (73.11)	480 (75.12)	99 (64.71)		
Previous surgical history, *n* (%)				*χ*^2^ = 0.80	0.372
No	477 (60.23)	380 (59.47)	97 (63.40)		
Yes	315 (39.77)	259 (40.53)	56 (36.60)		
Imaging reports of hydronephrosis, *n* (%)				*χ*^2^ = 10.95	**<**.**001**
No	196 (24.75)	174 (27.23)	22 (14.38)		
Yes	596 (75.25)	465 (72.77)	131 (85.62)		

*Z*, Mann–Whitney test; *χ*^2^, Chi-square test.

M, Median; Q_1_, 1st Quartile; Q_3_, 3rd Quartile.

Bold values indicate statistical significance (*P* < 0.05).

Significant differences between the two groups were observed in age, stone size, duration of stone history, and hydronephrosis. Patients in the failure group had smaller stones than those in the success group, with a median major axis of 8.84 mm (IQR, 7.04–12.45) vs. 11.55 mm (IQR, 8.79–15.01) (*P* < 0.001), and a median minor axis of 6.00 mm (IQR, 4.13–7.83) vs. 8.12 mm (IQR, 6.28–10.18) (*P* < 0.001).

Stone history of ≤31 days and hydronephrosis were more frequent in the failure group. No significant differences were found in height, BMI, CT value, sex, smoking history, drinking history, hypertension, diabetes, coronary heart disease, chronic kidney disease, stone side, stone location, number of stones, previous surgical history, or prior history of UAS insertion failure.

### Risk-factor analysis

Univariable and multivariable logistic regression analyses are shown in Table 2. In multivariable analysis, stone history >31 days was associated with a lower risk of UAS insertion failure (OR = 0.61, 95% CI: 0.41–0.92, *P* = 0.017), whereas hydronephrosis (OR = 1.79, 95% CI: 1.07–2.99, *P* = 0.027), age (OR = 1.02, 95% CI: 1.01–1.03, *P* = 0.019), and larger major stone axis (OR = 1.13, 95% CI: 1.06–1.20, *P* < 0.001) were associated with increased risk. In contrast, larger minor stone axis was associated with a lower risk of failure (OR = 0.64, 95% CI: 0.57–0.73, *P* < 0.001). Proximal ureteral stone location was significant in univariable analysis but not after multivariable adjustment. Although the major and minor stone axes were strongly correlated (*r* = 0.814), multicollinearity assessment showed low adjusted VIF values for the variables included in the multivariable model (Supplementary Table 2). In an additional subgroup analysis restricted to patients with ureteral stones, minor axis remained negatively associated with failure, whereas major axis was no longer significant after adjustment (Supplementary Table 3).

**Table 2:** Logistic regression.

Variables	Unilogistic					Multilogistic				
	*β*	S.E	*Z*	*P*	OR (95%CI)	*β*	S.E	*Z*	*P*	OR (95%CI)
Sex										
Female					1.00 (Reference)					
Male	0.14	0.21	0.66	0.508	1.15 (0.76–1.74)					
Smoking history										
No					1.00 (Reference)					
Yes	0.38	0.22	1.79	0.074	1.47 (0.96–2.24)					
Drinking history										
No					1.00 (Reference)					
Yes	−0.17	0.27	−0.63	0.527	0.84 (0.50–1.43)					
Hypertension										
No					1.00 (Reference)					
Yes	0.34	0.19	1.80	0.072	1.40 (0.97–2.03)					
Diabetes										
No					1.00 (Reference)					
Yes	0.31	0.26	1.18	0.237	1.37 (0.82–2.29)					
Coronary heart disease										
No					1.00 (Reference)					
Yes	0.41	0.53	0.78	0.437	1.51 (0.53–4.25)					
Chronic kidney disease										
No					1.00 (Reference)					
Yes	0.59	0.70	0.85	0.396	1.81 (0.46–7.06)					
Stone side										
Left					1.00 (Reference)					
Right	−0.19	0.19	−1.00	0.318	0.83 (0.57–1.20)					
Both	0.48	0.31	1.57	0.117	1.62 (0.89–2.94)					
Stone location										
Renal area					1.00 (Reference)					1.00 (Reference)
Proximal ureter	0.54	0.21	2.61	**0**.**009**	1.72 (1.14–2.58)	0.14	0.23	0.62	0.534	1.15 (0.74–1.80)
Mid ureter	0.37	0.31	1.20	0.232	1.45 (0.79–2.66)	−0.16	0.34	−0.46	0.643	0.86 (0.44–1.65)
Distal ureter	0.30	0.35	0.86	0.389	1.35 (0.68–2.67)	−0.13	0.38	−0.35	0.727	0.88 (0.41–1.85)
Number of stones										
Single					1.00 (Reference)					
Multiple	0.24	0.18	1.28	0.200	1.27 (0.88–1.82)					
UAS insertion failure history										
No					1.00 (Reference)					
Yes	−1.12	0.61	−1.85	0.064	0.33 (0.10–1.07)					
Stone history length										
≤31 Days					1.00 (Reference)					1.00 (Reference)
>31 Days	−0.50	0.19	−2.59	**0**.**010**	0.61 (0.42–0.89)	−0.49	0.21	−2.38	**0**.**017**	0.61 (0.41–0.92)
Previous surgical history										
No					1.00 (Reference)					
Yes	−0.17	0.19	−0.89	0.373	0.85 (0.59–1.22)					
Imaging reports of hydronephrosis										
No					1.00 (Reference)					1.00 (Reference)
Yes	0.80	0.25	3.24	**<**.**001**	2.23 (1.37–3.62)	0.58	0.26	2.21	**0**.**027**	1.79 (1.07–2.99)
Height	−0.01	0.01	−0.83	0.408	0.99 (0.97–1.01)					
BMI	−0.04	0.03	−1.36	0.173	0.96 (0.91–1.02)					
Age	0.02	0.01	2.21	**0**.**027**	1.02 (1.01–1.03)	0.02	0.01	2.34	**0**.**019**	1.02 (1.01–1.03)
Major axis of stones	−0.10	0.02	−4.52	**<**.**001**	0.91 (0.87–0.95)	0.12	0.03	3.57	**<**.**001**	1.13 (1.06–1.20)
Minor axis of calculus	−0.29	0.04	−7.58	**<**.**001**	0.75 (0.70–0.81)	−0.44	0.06	−7.03	**<**.**001**	0.64 (0.57–0.73)
CT value	−0.00	0.00	−0.52	0.601	1.00 (1.00–1.00)					

OR, odds ratio; CI, confidence interval.

Bold values indicate statistical significance (*P* < 0.05).

Given the opposite directions of the major and minor stone axes in the multivariable model, sensitivity analyses were performed using alternative stone-size parameterizations (Supplementary Table 4). When modeled separately, both the major and minor axes were associated with a lower risk of UAS insertion failure, while a higher aspect ratio was associated with increased risk.

### Exploratory model comparison and SHAP-based feature interpretation

**Figure 1:**
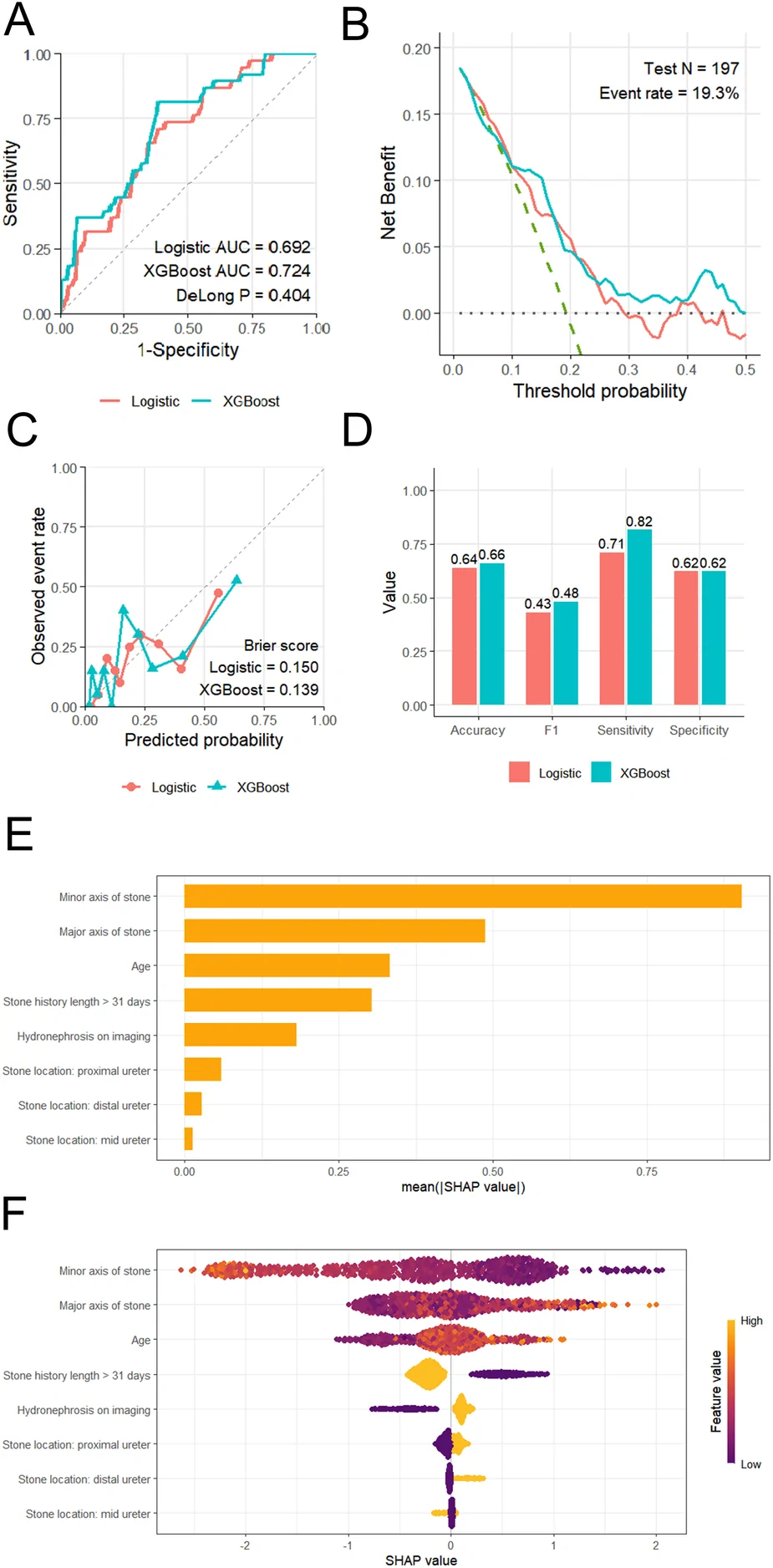
Performance of the prediction models for ureteral access sheath insertion failure. **(A)** Receiver operating characteristic (ROC) curves comparing model discrimination. **(B)** Decision curve analysis. **(C)** Calibration curves showing agreement between predicted and observed probabilities. **(D)** Comparison of performance metrics, including accuracy, F1 score, sensitivity, and specificity. **(E)** Mean absolute SHAP value plot showing the average importance of each predictor. **(F)** Beeswarm plot illustrating the direction and magnitude of feature contributions. Positive SHAP values indicate increased predicted risk, whereas negative values indicate decreased risk. Each dot represents an individual patient.

To further characterize the predictive contribution of the identified clinical factors, XGBoost and a logistic regression comparator were developed using the same candidate predictors and evaluated in the same test cohort. ROC analysis showed similar discrimination between XGBoost and logistic regression, with AUCs of 0.724 and 0.692, respectively, and no significant difference by the DeLong test (*P* = 0.404; Figure 1A). Decision curve analysis and calibration curves also showed broadly comparable performance between the two models (Figures 1B,C). Threshold-dependent performance metrics are summarized in Figure 1D.

SHAP analysis was used for feature-level interpretation of the XGBoost model. The mean absolute SHAP value plot summarized the relative contribution of included predictors (Figure 1E), while the beeswarm plot further showed their direction of effect (Figure 1F). Shorter stone history, hydronephrosis, proximal ureteral stone location, and specific stone-size patterns generally contributed to a higher predicted probability of UAS insertion failure, broadly consistent with the logistic regression-based findings.

### Systematic review and meta-analysis

During the literature search, we noted that the included studies targeted two distinct patient populations. The first comprised individuals with a “primary ureter,” defined as patients who may have experienced ureteral stones previously but had never undergone any acute stone surgery, leaving their ureter in an intervention-naïve state. The second group did not exclude patients with a history of prior ureteral interventions. On this basis, we conducted a subgroup analysis. The meta-analysis incorporated 10 studies published between 2015 and 2024, spanning across five countries: the United States, Japan, China, Canada, and Turkey. Our own collected data were also included as one of the studies in the meta-analysis (except for age, which was excluded due to the non-normal distribution).

**Figure 2:**
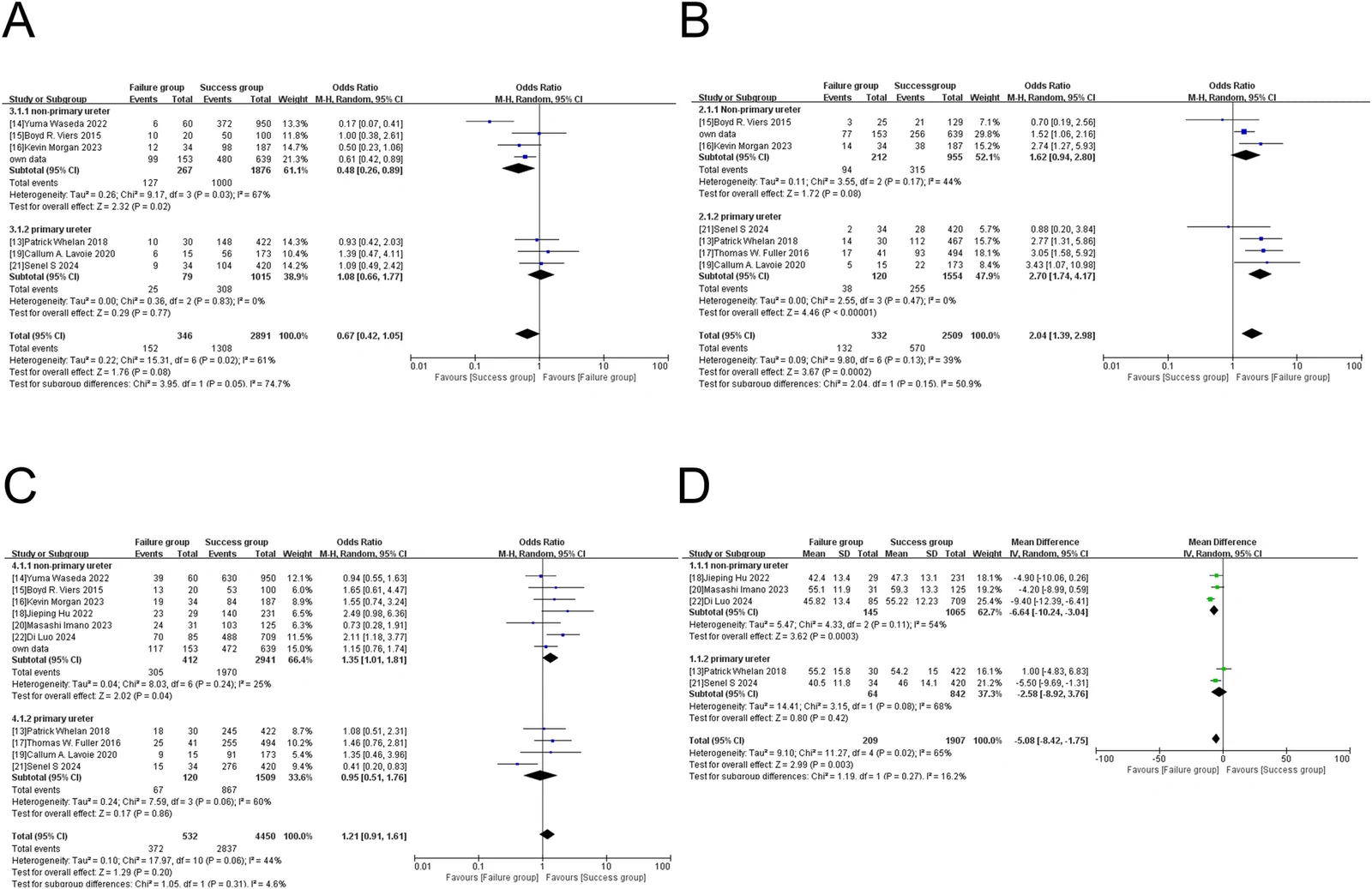
Forest plots of the meta-analysis. Subgroup analyses were performed according to study population characteristics. “Primary ureter” was defined as patients without prior acute ureteral stone surgery. **(A)** Association between stone history >31 days and UAS insertion failure. **(B)** Association between proximal ureteral stone location and UAS insertion failure. **(C)** Association between male sex and UAS insertion failure. **(D)** Comparison of age between the failure and success groups.

Stone history >31 days (9 studies, *n* = 3,237) was not significantly associated with failure (OR = 0.67, 95% CI: 0.42–1.05, *P* = 0.08). However, subgroup analysis revealed a significant association in the non-primary ureter group (OR = 0.48, *P* = 0.02). Stones located in the proximal ureter (7 studies, *n* = 2,841) were significantly more frequent in the failure group (OR = 2.04, 95% CI: 1.39–2.98, *P* < 0.01), with subgroup effects stronger in the primary ureter group (OR = 2.70, *P* < 0.01). For male sex (10 studies, *n* = 4,982), no significant association was found overall (OR = 1.21, *P* = 0.20) or in subgroup analysis. The direction of the age association differed between the cohort analysis and meta-analysis. In the meta-analysis, age was lower in the failure group (5 studies, *n* = 2,116; MD = −5.08 years, 95% CI: −8.42 to −1.75, *P* < 0.01), whereas older age was positively associated with failure in the cohort-based multivariable logistic regression (Figure 2). A flowchart of the literature review is presented in Supplementary Figure 1. The studies included are presented in Supplementary Table 5.

Quality assessment and leave-one-out sensitivity analyses are provided in Supplementary Tables 6, 7. Sensitivity analyses supported the robustness of the association between proximal ureteral stone location and UAS insertion failure, whereas the pooled estimates for previous stone history and sex were less stable.

## Discussion

In this multicenter cohort study, UAS insertion failure occurred in 19.3% of patients undergoing retrograde intrarenal surgery with attempted 11/13F UAS placement. Multivariable logistic regression identified stone history length, hydronephrosis, age, and stone-size parameters as factors associated with failure. XGBoost and SHAP analyses provided complementary feature-level interpretation, while the meta-analysis synthesized previous evidence and supported the relevance of proximal ureteral stone location. The association with age was less consistent between the cohort and meta-analysis.

Preoperative ureteral stenting has been reported to reduce the risk of UAS insertion failure (24). However, routine preoperative stent placement is not currently recommended in clinical guidelines (2). Pharmacological strategies, including preoperative *α*1-blocker administration, have also been investigated as potential approaches to facilitate UAS placement (25, 26). In the present cohort, routine elective pre-stenting before the index UAS placement attempt was not performed. Patients who required temporary double-J stent placement after failed UAS insertion were classified as UAS insertion failure cases, rather than as a separate pre-stented group. Surgical background was partly captured by previous surgical history, which was not significantly associated with UAS insertion failure in logistic regression analysis.

Previous studies have reported that stone size is associated with ureteral access sheath placement, and smaller stone diameter has a higher risk of difficult ureter (22, 23). Stone-size parameters possibly were associated with UAS insertion failure, but the directions of the major and minor axes differed in our cohort multivariable model. To further explore this finding, we performed sensitivity analyses using alternative stone-size parameterizations. When modeled separately, both stone axes were negatively associated with failure, whereas aspect ratio was positively associated with failure. Then we further performed a subgroup analysis restricted to ureteral stones. In this subgroup, minor axis remained negatively associated with failure, whereas major axis was no longer significant after adjustment. This may suggest that, for ureteral stones, the transverse stone dimension may more closely reflect the relationship between the stone and the ureteral lumen, although this interpretation remains exploratory (27).

Other clinical factors showed generally consistent patterns across the cohort analysis, SHAP interpretation, and meta-analysis. Stone history >31 days was negatively associated with UAS insertion failure, whereas hydronephrosis was positively associated with failure, with similar directions observed in SHAP analysis. Age should be interpreted cautiously because the effect size in the cohort analysis was modest and only a limited number of studies reported age in the meta-analysis. Proximal ureteral location showed a risk-increasing pattern in univariable analysis, SHAP analysis, and the meta-analysis. However, it was not statistically significant in multivariable logistic regression, possibly due to adjustment for stone-axis measurements.

Hydronephrosis on imaging indicates the severity of obstruction and structural changes of the ureter (28). Stone history length may reflect multiple clinical factors, including disease stage, obstruction status, and symptom duration. Considering previous study definitions and potential recall bias in electronic medical records, we did not model stone history length as an exact continuous variable. Although there exist anatomical differences between male and female ureters, and Jieping Hu's study reported such distinctions, the overall evidence remains insufficient to support sex as an independent influencing factor (19, 29, 30).

SHAP values reflect the contribution of each feature to prediction within the XGBoost model, whereas multivariable logistic regression estimates the adjusted statistical association between each variable and the outcome. Thus, SHAP-based importance may provide complementary information but requires cautious interpretation when it differs from multivariable regression results. In the present study, SHAP analysis provided feature-level visualization of the relative contribution and direction of included predictors within the XGBoost model. Image-analysis-based algorithms have been explored to improve predictive performance in other fields (31, 32). A recent review has summarized the mechanical characteristics of the ureter and their relationships with anatomical location, patient age, and endourological manipulation, highlighting the complex biomechanical properties of the ureter that may influence the success of ureteral access sheath placement (33, 34). The observed patterns for stone-size parameters suggested that stone-related factors should not be evaluated solely by maximal diameter or anatomical location. Future studies incorporating more detailed anatomical, biomechanical, endoscopic, or image-derived features may provide a more comprehensive assessment of UAS insertion difficulty (35–38).

This study has several strengths. First, multivariable logistic regression was used as the primary risk-factor analysis, while SHAP analysis and meta-analysis provided complementary evidence from model-based interpretation and previously published studies. This integrated framework strengthened the interpretation of factors associated with UAS insertion failure. Second, the multicenter design improved the clinical representativeness of the cohort and reduced the likelihood that the findings were driven by a single institution. Third, the study population was not restricted to patients with a primary ureter, thereby better reflecting real-world clinical practice. Finally, additional analyses, including multicollinearity assessment, alternative stone-size parameterization, and ureteral-stone subgroup analysis, were performed to address the complex associations between stone-size parameters and UAS insertion failure.

Several limitations should be acknowledged. First, all included patients underwent attempted placement of an 11/13F UAS. Because 11/13F is not the smallest available sheath size, some patients classified as insertion failure might have achieved successful access if a smaller sheath had been attempted. This may partly explain the relatively high failure rate in the present cohort and limits direct extrapolation of the findings to procedures using different sheath-size strategies. Second, UAS insertion is operator-dependent, but detailed information on individual surgeon experience, annual procedural volume, center-specific case volume, and sheath insertion technique was not available. Therefore, residual confounding related to surgeon- and center-level practice patterns cannot be excluded. Third, the predictive performance of the models was moderate, and further improvement is required before clinical implementation. Future studies incorporating more detailed anatomical, biomechanical, endoscopic, or image-derived features may improve risk assessment. Several limitations of the meta-analysis should be acknowledged. First, the systematic review was not prospectively registered in PROSPERO, which may limit transparency of the review process. Second, grey literature was not systematically searched, and only English-language studies were included; therefore, publication and language bias could not be fully excluded. In addition, because fewer than 10 studies were included in each pooled analysis, formal assessment of publication bias was not performed.

## Conclusions

UAS insertion failure was associated with hydronephrosis, stone-size parameters, and stone history length, with age requiring cautious interpretation. Proximal ureteral stone location may provide additional clinical information when interpreted together with other stone-related factors. SHAP-based visualization and meta-analysis offered complementary evidence to the regression-based findings. These results may support individualized preoperative assessment before attempted placement of a planned 11/13F UAS.

## Data Availability

The raw data supporting the conclusions of this article will be made available by the authors, without undue reservation.
