# Supplementary material for: Clinical risk factors and machine learning-assisted interpretation of ureteral access sheath insertion failure: a multicenter study and meta-analysis
Source: Front Surg. 2026 Aug 26;13:1871236. doi: 10.3389/fsurg.2026.1871236 (PMC13553799; doi:10.3389/fsurg.2026.1871236)
Supplement: Supplementary file 1 [file Table1.docx]

**Supplementary Table 1. Case distribution and UAS insertion failure rates across participating centers**

| **Center** | **Total cases** | **Failure cases** | **Failure rate** |
| --- | --- | --- | --- |
| The First Affiliated Hospital of Nanjing Medical University | 281 | 53 | 18.8% |
| The Second Affiliated Hospital of Nanjing Medical University | 135 | 25 | 18.5% |
| Yixing People's Hospital | 74 | 15 | 20.3% |
| Gaoyou People's Hospital | 99 | 18 | 18.2% |
| Huai'an First People's Hospital | 82 | 17 | 20.7% |
| The Affiliated Shuyang Hospital of Xuzhou Medical University | 121 | 25 | 20.7% |

**Failure rate was calculated as the number of UAS insertion failure cases divided by the total number of included cases in each center. UAS,(ureteral access sheath).**
