# Supplementary material for: Clinical risk factors and machine learning-assisted interpretation of ureteral access sheath insertion failure: a multicenter study and meta-analysis
Source: Front Surg. 2026 Aug 26;13:1871236. doi: 10.3389/fsurg.2026.1871236 (PMC13553799; doi:10.3389/fsurg.2026.1871236)
Supplement: Supplementary file 2 [file Table2.docx]

## **Supplementary Table 2. Correlation between stone size parameters**

**A. Correlation between stone-size parameters**

| Variables | Major axis | Minor axis |
| --- | --- | --- |
| Major axis | 1.000 | 0.814 |
| Minor axis | 0.814 | 1.000 |

**B. Multicollinearity assessment using variance inflation factors**

| **Variable** | **GVIF** | **Df** | **Adjusted VIF** |
| --- | --- | --- | --- |
| Stone location | 1.113 | 3 | 1.018 |
| Stone history length | 1.017 | 1 | 1.008 |
| Major axis of stones | 2.707 | 1 | 1.645 |
| Minor axis of stones | 2.752 | 1 | 1.659 |
| Hydronephrosis | 1.026 | 1 | 1.013 |
