# Supplementary material for: Clinical risk factors and machine learning-assisted interpretation of ureteral access sheath insertion failure: a multicenter study and meta-analysis
Source: Front Surg. 2026 Aug 26;13:1871236. doi: 10.3389/fsurg.2026.1871236 (PMC13553799; doi:10.3389/fsurg.2026.1871236)
Supplement: Supplementary file 3 [file Table3.docx]

**Supplementary Table 3. Subgroup analysis restricted to patients with only ureteral stones**

| Variables | β | S.E | Z | *P* | OR (95%CI) |
| --- | --- | --- | --- | --- | --- |
|  |  |  |  |  |  |
| Intercept | -0.79 | 0.69 | -1.15 | 0.252 | 0.46 (0.12 ~ 1.75) |
| Imaging-reports-of-hydronephrosis |  |  |  |  |  |
| 0 |  |  |  |  | 1.00 (Reference) |
| 1 | 0.81 | 0.40 | 2.04 | **0.041** | 2.24 (1.03 ~ 4.87) |
| Age | 0.02 | 0.01 | 2.28 | **0.023** | 1.02 (1.01 ~ 1.04) |
| Minor-axis-of-calculus | -0.35 | 0.08 | -4.20 | **<.001** | 0.70 (0.60 ~ 0.83) |
| Major-axis-of-stones | 0.00 | 0.05 | 0.08 | 0.936 | 1.00 (0.91 ~ 1.11) |
| OR: Odds Ratio, CI: Confidence Interval | | | | | |
