# Supplementary material for: Clinical risk factors and machine learning-assisted interpretation of ureteral access sheath insertion failure: a multicenter study and meta-analysis
Source: Front Surg. 2026 Aug 26;13:1871236. doi: 10.3389/fsurg.2026.1871236 (PMC13553799; doi:10.3389/fsurg.2026.1871236)
Supplement: Supplementary file 4 [file Table4.docx]

**Supplementary Table 4. Sensitivity analyses using alternative stone-size parameterizations in multivariable logistic regression**

| **Model** | **Stone-size parameterization** | **Stone variable** | **OR (95% CI)** | **P value** | **Interpretation** |
| --- | --- | --- | --- | --- | --- |
| Main model | Major axis + minor axis | Major axis | 1.13 (1.06–1.20) | <0.001 | Major axis adjusted for minor axis |
| Main model | Major axis + minor axis | Minor axis | 0.64 (0.57–0.73) | <0.001 | Minor axis adjusted for major axis |
| Sensitivity model 1 | Major axis only | Major axis | 0.91 (0.87–0.95) | <0.001 | Isolated association of long-axis stone size |
| Sensitivity model 2 | Minor axis only | Minor axis | 0.75 (0.70–0.81) | <0.001 | Isolated association of short-axis stone size |
| Sensitivity model 3 | Stone area only | Stone area | 0.99 (0.99–0.99) | <0.001 | Overall stone burden |
| Sensitivity model 4 | Aspect ratio only | Aspect ratio | 2.37 (1.60–3.52) | <0.001 | Stone morphology elongation |

The main model included both major and minor stone axes simultaneously. Sensitivity models evaluated alternative parameterizations of stone size. All models were adjusted for the same covariates selected in the cohort-based multivariable logistic regression, except that the stone-size variables were replaced as specified. Stone area was calculated as major axis × minor axis, and aspect ratio was calculated as major axis / minor axis. OR, odds ratio; CI, confidence interval.
