# Supplementary material for: Clinical risk factors and machine learning-assisted interpretation of ureteral access sheath insertion failure: a multicenter study and meta-analysis
Source: Front Surg. 2026 Aug 26;13:1871236. doi: 10.3389/fsurg.2026.1871236 (PMC13553799; doi:10.3389/fsurg.2026.1871236)
Supplement: Supplementary file 5 [file Table5.docx]

**Supplementary Table 5**

**Study Characteristics of the Included Articles in the Systematic Review With Meta-analysis**

| **No** | **Year** | **country** | **Study design** | **Analytical method** | **Samole select** | **Sample size(failure/success)** | **Risk factor** |
| --- | --- | --- | --- | --- | --- | --- | --- |
| [1] | 2018 | America | case–control study | Logistic regression | primary ureteroscopies | 30/422 | Stone location(Proximal ureter) |
| [2] | 2022 | Japan | case–control study | Logistic regression | nom-primary ureteroscopies | 60/950 | Age  Renal stone group Stone history |
| [3] | 2015 | America | case–control study | logistic regression | non-primary ureteroscopies | 25/129 | Prior ipsilateral surgery Prior ipsilateral stent |
| [4] | 2023 | America | case–control study | logistic regression | non-primary ureteroscopies | 34/187 | Stone location（Proximal ureter） Previous stone passage |
| [5] | 2021 | Canada | case–control study | Chi-squared and t-test analysis | primary ureteroscopies | 15/173 | Stone location（Proximal ureter） Time from first diagnosis to URS |
| [6] | 2022 | China | case–control study | logistic regression | non-primary ureteroscopies | 29/231 | Sex Diameter of the ipsilateral Common iliac artery Length of history |
| [7] | 2016 | America | case–control study | logistic regression | primary ureteroscopies | 41/494 | Stone location(Proximal ureter) Young female population |
| [8] | 2024 | Turkey | case–control study | logistic regression | primary ureteroscopies | 34/420 | Prior urinary tract infection Age. |
| [9 | 2024 | China | case–control study | Lasso combined logistic regression | non-primary ureteroscopies | 85/709 | Age  History of lithotripsy Sex BMI Diameter of ureteral stone |
| [10] | 2023 | Japan | case–control study | logistic regression | non-primary ureteroscopies | 31/125 | Major axis <8mm Negative traceability Smoking status Diabetes mellitus |
|  |  |  |  |  |  |  |  |
