# Supplementary material for: Clinical risk factors and machine learning-assisted interpretation of ureteral access sheath insertion failure: a multicenter study and meta-analysis
Source: Front Surg. 2026 Aug 26;13:1871236. doi: 10.3389/fsurg.2026.1871236 (PMC13553799; doi:10.3389/fsurg.2026.1871236)
Supplement: Supplementary file 6 [file Table6.docx]

**Supplementary Table 6. Quality assessment of included studies(NOS）**

| **No.** | **Study** | **Selection** | **Comparability** | **Exposure** | **Total score** | **Quality** |
| --- | --- | --- | --- | --- | --- | --- |
| 14 | Whelan et al., 20182 | 4 | 1 | 2 | 7 | High |
| 15 | Waseda et al., 2022 | 4 | 2 | 2 | 8 | High |
| 16 | Viers et al., 2015 | 4 | 1 | 3 | 8 | High |
| 17 | Morgan et al., 2023 | 4 | 1 | 2 | 7 | High |
| 18 | Lavoie et al., 2021 | 4 | 1 | 2 | 7 | High |
| 19 | Hu et al., 2022 | 4 | 2 | 2 | 8 | High |
| 20 | Fuller et al., 2016 | 4 | 1 | 2 | 7 | High |
| 21 | Senel et al., 2024 | 4 | 1 | 2 | 7 | High |
| 22 | Luo et al., 2024 | 4 | 2 | 2 | 8 | High |
| 23 | Imano et al., 2023 | 4 | 2 | 2 | 8 | High |
