# Supplementary material for: Clinical risk factors and machine learning-assisted interpretation of ureteral access sheath insertion failure: a multicenter study and meta-analysis
Source: Front Surg. 2026 Aug 26;13:1871236. doi: 10.3389/fsurg.2026.1871236 (PMC13553799; doi:10.3389/fsurg.2026.1871236)
Supplement: Supplementary file 7 [file Table7.docx]

**Supplementary table 7 Leave-one-out sensitivity analyses for pooled risk factors in the meta-analysis**

| **Risk factor** | **Excluded study** | **Effect estimate after exclusion** | **P** | **I²** |
| --- | --- | --- | --- | --- |
| Proximal ureteral stone | [15]Boyd R. Viers 2015 | 2.18 [1.52, 3.12] | <0.001 | 32% |
|  | [16]Kevin Morgan 2023 | 1.93 [1.25, 2.98] | 0.003 | 43% |
|  | Present cohort | 2.34 [1.52, 3.59] | <0.001 | 23% |
|  | [13]Patrick Whelan 2018 | 1.92 [1.23, 3.00] | 0.004 | 42% |
|  | [17]Thomas W. Fuller 2016 | 1.86 [1.22, 2.84] | 0.003 | 33% |
|  | [19]Callum A. Lavoie 2020 | 1.94 [1.28, 2.94] | 0.001 | 43% |
|  | [21]Senel S 2024 | 2.14 [1.45, 3.18] | <0.001 | 42% |
| Stone history | [14]Yuma Waseda 2022 | 0.73 [0.56, 0.96] | 0.020 | 1% |
|  | [15]Boyd R. Viers 2015 | 0.63 [0.38, 1.05] | 0.070 | 65% |
|  | [16]Kevin Morgan 2023 | 0.70 [0.41, 1.20] | 0.200 | 66% |
|  | Present cohort | 0.69 [0.37, 1.27] | 0.230 | 68% |
|  | [13]Patrick Whelan 2018 | 0.63 [0.38, 1.06] | 0.080 | 65% |
|  | [19]Callum A. Lavoie 2020 | 0.61 [0.38, 0.98] | 0.040 | 62% |
|  | [21]Senel S 2024 | 0.61 [0.37, 1.01] | 0.060 | 63% |
|  |  |  |  |  |
| Gender | [14]Yuma Waseda 2022 | 1.25 [0.91, 1.72] | 0.170 | 47% |
|  | [15]Boyd R. Viers 2015 | 1.18 [0.87, 1.60] | 0.280 | 49% |
|  | [16]Kevin Morgan 2023 | 1.18 [0.86, 1.61] | 0.300 | 48% |
|  | [18]Jieping Hu 2022 | 1.15 [0.86, 1.53] | 0.350 | 42% |
|  | [20]Masashi Imano 2023 | 1.25 [0.93, 1.69] | 0.150 | 47% |
|  | [22]Di Luo 2024 | 1.12 [0.84, 1.48] | 0.440 | 35% |
|  | Present cohort | 1.22 [0.87, 1.71] | 0.250 | 50% |
|  | [13]Patrick Whelan 2018 | 1.22 [0.89, 1.67] | 0.210 | 50% |
|  | [17]Thomas W. Fuller 2016 | 1.18 [0.86, 1.62] | 0.300 | 49% |
|  | [19]Callum A. Lavoie 2020 | 1.20 [0.88, 1.63] | 0.240 | 50% |
|  | [21]Senel S 2024 | 1.32 [1.07, 1.63] | 0.010 | 0% |
| Age | [18]Jieping Hu 2022 | -5.00 [-9.14, -0.87] | 0.020 | 73% |
|  | [20]Masashi Imano 2023 | -5.18 [-9.30, -1.06] | 0.010 | 72% |
|  | [22]Di Luo 2024 | -3.83 [-6.46, -1.21] | 0.004 | 13% |
|  | [13]Patrick Whelan 2018 | -6.50 [-9.16, -3.84] | <0.001 | 39% |
|  | [21]Senel S 2024 | -4.80 [-9.20, -0.40] | 0.030 | 73% |
